# Abstracts and Gleanings

**Published:** 1878-06-20

**Authors:** 


### *ABSTRACTS AND GLEANINGS.*

### TREATMENT OF PILES.

This must be considered under two heads: first, remove predisposing cause; second, remove the disease.

1. If the cause that produces the hemorrhoids is found, it should be removed. Persons whose habits of life predispose them to piles should be instructed to keep their bowels regular by a judicious regulation of the diet. When medicines are needed, I am in the habit of prescribing a pill composed of one-fourth grain each of aloes, podophyllin, and extract of belladonna, and one-twentieth grain of strychnine—one pill to be taken at night when needed. The use of tannin suppositories is also beneficial in the earlier stages, when only a slight enlargement of the veins exists. In some cases, by this means, contraction of the blood-vessels, and radical cure for a time, have been brought about. If, however, the piles are well formed when we are called upon to treat them, only one means of cure exists, namely, surgical interference. Salves, lotions, etc., give but temporary relief; we should urge our patient to submit to some surgical operation.

2. Removal of piles is accomplished in various ways. External piles can be simply snipped off with the scissors, as hemorrhage need not be feared. Internal piles can be cauterized by applying fuming nitric acid and returning them into the bowel; a slough forms, which is cast off in a few days.

Lately, a plan has received very favorable attention and extensive trial: it is to inject into the piles carbolic acid, diluted more or less (generally, one part of acid to three of glycerine). The piles are each injected with three to ten drops of the acid and returned into the bowel; sloughing takes place in a few days, a cure being effected by destruction of the vessels.

These different procedures can be borne without the use of chloroform, and where the disease is not too far advanced will be sufficient to effect a cure. In chronic cases, and those where prolapsus exists, other means must be employed—ligation and the actual cautery are here indicated. Mode of procedure: The bowels should be emptied by a cathartic and washed out thoroughly with a syringe; the patient should be put under the influence of an anæsthetic, as the pain and resulting contraction of the sphincter interfere with the operation; the piles should be pulled down; in a woman, this is easy by putting a finger in the vagina; but in a man, considerable difficulty is sometimes experienced. I saw, in a German work, a plan recommended that seemed very good: it is to introduce into the rectum a good-sized sponge, to which a string is attached; the sponge can be introduced by pressing it together; it expands when in the rectum, and, by drawing on the string, the piles are pulled out before the sponge; when the piles are all out, the small ones are ligated simply, silk, cat-gut or rubber being used. The ligature must be drawn tight, so that the vessels are entirely closed. The larger flat or oblong piles should be transfixed by a threaded needle and tied on each side; sometimes the mucous membrane around the base of the pile is cut through to the submucous tissue to prevent the ligature from slipping. The ligatures are cut short, and the whole mass of piles returned to the bowel; the ligated parts slough off, and a permanent cure is generally effected in a week or ten days.

The relaxation of the sphincter ani, spoken of before, is one of the greatest difficulties to overcome, even if the piles are obliterated. Probably the most success can be effected by the plan lately proposed by Voillemer. By the actual or galvano-cautery, linear cauterizations are produced in two, three or four places, the resulting cicatrix producing such a contraction that prolapsus does not take place any more.—Dr. Carstens, *in Detroit Lancet.*

---

THE STRONG ELASTIC BANDAGE.

The treatment of varicose and other chronic ulcers of the leg is so generally unsatisfactory, that any new method promising favorable results is to be hailed with delight.

The latest novelty is the use of the strong elastic bandage, with which Dr. Henry Martin claims to have cured over six hundred cases without a single failure. The bandage is of "pure rubber," ten and a half feet along, three inches wide, and thickness of No. 21 "Stubs' wire guage." The length and breadth may vary with the size of the limb, but this is the most desirable thickness. It is applied by winding one turn just above the malleoli, then one around the instep and sole, then spirally up the leg to the knee, where it is fastened by tapes attached to the end of the bandage for that purpose. If it is desirable to apply it as far as the groin, a bandage eighteen to tweenty feet long will be necessary: At night the bandage is removed and the ulcer protected by a piece of oiled linen, or some equally simple dressing. In the morning all traces of oil or cerates must be carefully removed, as fatty matters tend to injure the rubber, and the bandage should be reapplied before leaving the bed. It should be applied with just sufficient snugness to prevent it slipping down, and the increase of blood in the veins on standing will cause it to become of the exact degree of tightness. The bandage keeps the leg warm, moist and air-tight, conditions most favorable to granulation and cicatrization, and in addition the gentle, even pressure so supports the distended and weakened vascular coats as to prevent that venous congestion so frequently the cause of the malnutrition of skin. For the first one or two weeks a papular eruption appears under the bandage caused by obstruction to the cutaneous follicles. The bandage is their best treatment. In non-specific ulcers no other local treatment is necessary. The circulation of the limb is not stopped, but, owing to the support given to the vessels, is facilitated; thus there need be no fear of causing œdema of the foot—on the contrary, the œdema which so constantly accompanies varicose ulcers is rapidly absorbed. The occurrence of œdema indicates the improper application of the bandage.

The use of this apparatus is not confined to the treatment of ulcers; injuries and diseases of the joints, especially of the knee and ankle, are equally benefitted. In sprains, the strong elastic bandage wound around a joint affords a constantly present substitute, externally, for the disabled ligament. The constant pressure induces a rapid absorption of the exudation among the tissues about the seat of injury, and the gentle, equable warmth and moisture, which always accompany its application, have a most favorable effect in alleviating and preventing inflammation. In diseases of the joints marked by effusion, the application of the bandage after aspiration, has been followed by complete success. In these cases the bandage should be applied day and night for six to eight weeks. Its use is also recommended in disease of bursæ mucosæ, œdema, erysipelas, and erythema, cutaneous affections, and as a redical cure for varicose veins; in the latter case it is supposed to act by cavsing adhesion of the walls of the vessels, and their consequent obliteration. *Med. Record.*

---

HOW TO DEPRIVE IODINE OF ITS STAIN.

(*Ex. Am. Jl. Med. Sciences.*) Add a few drops of carbolic acid to the tincture, and it will not stain; moreover, the tincture is more efficacious, and its action is more certain. M. Boggs recommends the following formula for use in injections: alcoholic tincture of iodine, three grammes; carbolic acid, six drops; glycerine, thirty grammes; distilled water, 150 grammes.

CHLORATE OF POTASH IN DIARRHŒA.

In the diarrhœa occurring most frequently in nervous and cachectic individuals, which consists of abundant and frequent serous discharges, which proves so obstinate to the usual treatment by astringents and narcotics; which is accompanied neither by increased pain on pressure over the lower part of the abdomen, nor by a coated tongue, and which, on post-mortem section, shows, as its single anatomical change, a slight redness of the intestinal mucosa, an Italian physician has found the chlorate of potash to be a valuable remedy. He calls it by this name because of his belief that its cause is a paralysis of the vasomotor nerves of the intestinal mucous membrane, which may in turn have its cause in insufficient nervous activity, due to disease of the affected nerve centers, or in a sympathy of the entire nervous system with the general cachexia. He thinks its essential nervous origin has been already demonstrated by the physiological observation, that after the removal of the cerebellum of animals, diarrhœa always supervenes.

This diarrhœa, according to the author of the paper, is the most frequent cause of death in Italian insane asylums; and it was only in those cases that had not yet reached a high grade of cachexia, that he was successful in averting a fatal termination by tonic and nourishing treatment. In most cases, treatment by the usual method was futile.

Having observed from the investigations of Sasse, that chlorate of potash seemed to increase the contractility of the muscular fibers in the vessel walls, he determined to test the agent in this disease. The result of his observations, although still requiring more extended tests, lead him to the following conclusions:

1. Chlorate of potash undoubtedly has a favorable effect upon vaso-paralytic diarrhœa. The result is often manifest as early as the first day of its administration.

2. To check the diarrhœa completely, it is usually necessary to continue it for several days, and to increase the dose according to the severity of the case.

3. If no improvement of the general bodily condition takes place during the time it is being given, the stoppage of the remedy is followed by a cessation of its favorable local effect. Its re-administration, however, again produces the usual good results.

4. In the severer cachexiæ, accompanied by great nervous depression, the action of this agent is slower; the diarrhœa is lessened, but not completely checked, and returns quite readily. In such cases we must resort to greatly increased doses. We must here recollect that the vaso-motor paralysis is of a high grade, or that organic changes (fatty or amyloid degeneration) have already taken place in the vessel walls, or changes in the mucous membrane (extravasations, ulcerations, etc.), which require an energetic and long-continued action of the agent to bring about a return to the normal condition.

5. This remedy is of little or no utility if diarrhœa be caused by active disease of the intestinal mucosa, (catarrhal enteritis, etc.)

6. It is also of service in senile diarrhœa, in that which precedes attacks of cholera, and in certain serous diarrhœa of warm climates. (One case of chronic diarrhœa contracted during a prolonged residence in Sicily, was cured by this plan).

7. The dose varies from 2 to 10 grammes (30-150 grains) per day, according to the severity of the illness.

---

ESMARCH ON CANCER.

In a recent lecture on cancer, Professor Esmarch said that he had frequently seen cancer originate upon a syphilitic basis, and often where the syphilis had been latent for a long period. He advised that cancers and malignant growths, wherever occurring, should be treated by arsenic and iodide of potassium internally and externally before proceeding to an operation.—*Maryland Med. Jour.*

KOUMISS.

As koumiss will not bear transportation to any considerable distance, it is desirable that the mode of preparing it should be generally known. I have therefore requested Mr. George I. McKelway, of Philadelphia, who has supplied me with all the koumiss my patients have used, to give the formula for its preparation. He writes as follows: "The manufacture of koumiss is a very easy and simple process. I take—

R.—Best unskimmed milk................ qt. j.
Yeast (brewers' or old bakers')...... grs. c.
Cane sugar........................ grs. cc.

"Keep the mixture at a temperature of 80° Fahr. until fermentation is quite brisk, stirring it frequently, and then bottle, carefully securing the corks with strong twine or wire. After twenty-four hours it is fit for use.

"The object of the addition of the cane sugar is the induction of alcoholic fermentation. If the sugar be left out the result is likely to be that lactic fermentation only is set up, and the product is sour milk. The quantity of sugar used has, of course, to be judged by the richness of the milk and its consequent richness in fermentable constituents."

The koumiss thus prepared by Mr. McKelway has proved entirely satisfactory. It is a very agreeable drink, having a slightly acid taste, and containing from three to four per cent. of alcohol, one to two per cent. of lactic acid, and is highly charged with carbonic acid gas. It contains the ordinary ingredients of milk, with the exception of the lactose (sugar of milk), most of which is converted into alcohol, lactic and carbonic acids. Koumiss is acid to litmus paper, both before and after being freed from carbonic acid. Its specific gravity is rather less than that of the milk from which it has been made (1.040 instead of 1.043). As it is important to retain its effervescing character, it should always be drawn by means of "champagne tap." It should be used within a few days of its preparation, since after two or three days the alcohol and lactic and carbonic acids increase so as to make it less agreeable and less well adapted to most cases. It should be kept on ice, or in a very cool place, as warmth soon causes the caseine to separate into a thick, heavy curd.

Koumiss may be said, then, to fairly represent the nutritive properties of good milk, while possessing, in addition, a mildly stimulating character. The carbonic acid gas with which it is highly charged, acts also as a sedative to the gastric mucous membrane, and thus renders it well adapted to cases where there is much irritability of stomach.—*Prof. Pepper, in Med. and Surg. Rep.*

---

PULSATILLA IN DYSMENORRHŒA.

A. B., 17 years old, came under our care November 24, 1876, for acne simplex of four years' standing. For several years has had disturbed menstruation, the periods not being equidistant and always preceded by severe pain, and often by hysterical attacks with slight convulsions. She has also profuse leucorrhœa continuing through the month. Treatment appropriate to her acne, together with tonics, was instituted.

Dec. 26.—Ordered tinct. of pulsatil. nuttal., two drops in water, an hour before meals. To commence its use three days before she expected her menses, and to discontinue it when any appeared.

Jan. 5.—Has menstruated since last visit, absolutely without pain or inconvenience. Continued pulsatilla twice daily.

Jan. 12.—Her leucorrhœa less troublesome. Continue pulsatilla once a day.

Jan. 25. — Has again menstruated without pain, but flowed for two days only.

April 28.—No dysmenorrhœa since last date. Have not seen her since. As the special treatment for her acne has no bearing upon the point under consideration, it is unnecessary to detail it.

DYSMENORRHŒA COMPLICATING ECZEMA.

C. D., 28 years old, married, came under my care March 12, 1877. Her eczema was of the dry, papular variety, situated chiefly about the mouth. For several years has suffered intensely at her monthly periods, the flow being scanty and not lasting more than twenty-four hours. Leucorrhœa moderate. Treatment ordered for the eczema only.

March 30.—Continued local and internal treatment for the eczema, and ordered her to take two drops of tinct. pulsatil. nuttal. for two days preceding her periods.

May 8.—Returned to-day and says that she took the pulsatilla as ordered, and that she did not suffer at all at her next period. After that she lost her medicine and did not take any before her last menstruation, which was last week. On this occasion she had a little but not much pain. She was re-supplied with the medicine. — *Dr. Piffard, in Med. Rec.*

### PROPHYLAXIS OF SCARLATINA.

Dr. S. H. Harmon accepts the germ theory as applied to all contagious diseases, and reasoning from that basis, that as we have specifics for many animal and vegetable parasites, there may be one for the peculiar germ causing scarlatina, he determined to try hyposulphite of soda, giving it to all the children of any family where the disease had appeared. He sums up the results as follows: In eight families he had forty-three children, of whom twenty-six contracted the disease, or sixty per cent. In every family the first was the only severe case, and almost always the last to get well: he did not have a single death. The dose of the hyposulphite was three-fourths of a grain of a grain of syrup for every year of age. This remedy is certainly worthy of a fair trial.

### ACTION OF ERGOT ON THE UTERUS.

Dr. C. C. McDowell, in Baltimore Medical Society, related a case of a woman who had never borne a child, to whom ergot had been given for the relief of hemorrhage. The administration of the drug was followed by violent expulsive pains. He related the case to sustain the opinion that ergot has an influence upon the unimpregnated uterus, and over that which never has been pregnant.

### ALUM IN AURAL DISCHARGES.

Dr. Chisolm, in North Carolina *Med. Journal*, says:

By means of alum, I have cured discharges of fifty years' standing in one week, and I now find very few aural discharges, however chronic,that withstands its proper application. The method employed in using it is first to thoroughly cleanse the ear, then wipe dry the passage by means of a loose cotton swab made at the end of a match or special applicator; after which, puff into the ear powdered alum, filling the drum-cavity with it. The very first application will often indicate a diminished discharge at the end of twenty-four hours. The ear is then washed out and the alum-powder again applied. This treatment is renewed once a day until the discharge is so reduced that the powder blown into the ear continues dry upon its exposed external surface. If it has crusted in the ear, it may be left for days as a hard mass, giving no pain and causing no annoyance. If, after a week or ten days' interval, the ear has seemingly stopped discharging, the alum-powder remaining dry, although in a cake, it may be syringed out, as if it were a foreign body. It usually leaves a healthy mucous membrane behind it.

Since powdered alum is so constantly and successfully used by me in aural discharges, I find it convenient to apply it through a puff-bottle, which expedites much the insufflation, and is far preferable to a quill or pipe-stem. In damp weather, I was formerly annoyed by the caking and lumping of the alum in the bottle, which necessitated frequent drying and repulverzation. I now add to it, at the suggestion of my assistant, Dr. W. A. McDowell, a small quantity of lycopodium powder, which, when thoroughly triturated with the alum, makes it more volatile, and not at all disposed to lump. Ten grains of lycopodium to the drachm of alum are ample.

In my own practice, I have ceased to consider chronic aural discharges an obstinate disease; for, under the thorough cleansing and the insufflation with alum, I find they yield more kindly to treatment than any other affection that has been of long continuance. One advantage of no small merit in the alum treatment is that it is incapable of abuse. An excessive application can do no harm.

### A STRANGE CASE.

'Tis an old saying that "truth is stranger than fiction;" and certainly the case I am about to relate is the strongest evidence of its truth. The case in question has reference to the little daughter of Mr. Samuel B., who resides in North East, Simpson county, Kentucky. As far as I know, both parents of the child are healthy, there being nothing in either to indicate the hereditary transmission of the disease. In March, 1877, she reached her fourth year, and at that time had attained the unprecedented weight, for that age, of one hundred pounds. She measures eighteen inches across the chest, and nearly five feet in height. Her mammæ were as fully developed as they are at puberty, and she menstruated regularly. Up to February, 1876, though, as shown above, she was remarkably developed, she had given no indication of the following strange phenomena. At that time her person became suddenly warmer than normal, and hair, soft and downy in color, like that of her head, commenced growing all over her body. In a short time it had completely covered her body with the exception of her face, palms of her hands and soles of her feet, and the skin was entirely hid from view. From the entire surface of her body there is a constant and profuse perspiration, of a very offensive odor, which is easy distinguishable at some distance from her. So profuse is it that in half an hour after being cleanly washed and dressed, her person and clothing will become saturated as thoroughly as if a bucket of water had been thrown over her. The perspiration is characteristic, being of a dark, yellow color, and of greater specific gravity than usual. Her voice is coarse like a man's, and sounds as though she was speaking in a barrel. Her strength is equal to that of a full grown man. Her intellect is much beyond her years. Her form is perfect. These things altogether go to make up the most wonderful case I ever heard or read of, and I think will be read with interest by every one. I will not attempt to account for its causation, but leave to the medical philosophers to solve the problem.— DR. ROBB, in Nashville *Jour. of Medicine and Surgery.*

### CARBUNCLE.

Dr. Crosthwaite (in *Med. Rep.*) relates three severe cases of carbuncle, treated with poultices and carbolic acid, as follows:

J. B. appeared at my office with a carbuncle on the dorsum of a middle finger, first phalanx. It was as large as could grow on the place, and so painful that he had not slept for several nights. He was forty-five years old, of good constitution, and fair health. I painted the surface of the sore freely with concentrated carbolic acid. Upon inquiring if the application gave him pain, he said no; that it felt better than it had felt for a week. Ordered poultices constantly, the caustic to be repeated on each change of poultice. Saw him a week afterward, and his carbuncle was gone and well, and he informed me that he had not a particle of pain after the first application of the remedy.

### TREATMENT OF ULCERS.

Dr. Mandelbaum, of Odessa, in a paper in a Berlin medical journal, asserts that all ulcers of the leg and other parts, whatever their character, age, and extent, can be cured by the following method. If they are very deep, with much loss of tissue, and with undermined, uneven, callous edges, they are first to be scraped away until healthy tissue is reached, with the modification of Volkmann's spoon as suggested by Hebra; they are then to be covered for several days with a thick layer of iodoform until fresh granulations spring up, (as they are certain to do), and until the base of the ulcer has reached the level of the surrounding skin. When this point in the healing process is reached, the ulcer is to be strapped daily with equal parts of mercurial and soap plaster of rather soft consistence, and carefully and evenly applied. Shallow ulcers which are covered only with a thick layer of pus require no preliminary scraping, and can be at once treated with iodoform, and, later on, strapped as above described.

---

### BEER AND SUICIDE.

According to the Quarterly Journal ot Inebriety, statistics indicate that most of the suicides following inebriety occur among beer-drinkers. The ultimate effect of lager beer, in many cases, is melancholy with a tendency to suicide. This is most prominent among the Germans, whose phlegmatic disposition is favorable to such a result. Beer has a peculiar psychological action, developing a low grade of depression in all cases.—*Boston Jour. of Chem.*

---

### NITRITE OF AMYL IN ASPHYXIA.

Nitrite of amyl, the nitrous ether of amylic alcohol, is a straw-colored liquid with a specific gravity of .877 and a boiling point between 93° and 99° C. It is a powerful stimulant of the heart, and its prompt action renders it of special value in cases of syncope and asphyxia. It has been used with success in the treatment of nervous headache, neuralgia, spasmodic asthma, and epilepsy. It is administered by inhalation, from one to five inspirations from the mouth of a vial being effectual.

In cases of apparent syncope from choke-damp, smoke, or mephitic exhalations, its use is also indicated, and in cases of apparent death from drowning it is likely to prove a potent auxiliary in resuscitation.

It is suggested that in cases of suspended respiration, when this agent cannot be employed in the usual manner, it may be conveyed to the stomach by the tube, or used hypodermically. That further experiments may be tried and the matter fully investigated is the object of this communication. — *Boston Jour. of Chem.*

---

### NEW METHOD OF DRESSING STUMPS.

Dr. Ed. Gaurreau, of Quebec, describes, in the *Lancet*, the method he adopts, as follows :

We shall suppose an amputation at the wrist. I apply the tourniquet over the brachial artery; I cut my flaps very carefully, that they may adjust as closely as possible, and I bring the divided parts together, and keep them in apposition by means of strips of linen one inch in width; soaked in a solution of equal parts of tincture of muriate of iron and water. I lay my strips first horizontally, and then spirally, using moderate and uniform pressure, so as to prevent subcutaneous oozing of blood, and I further saturate the compresses with iron. I now slightly turn the screw of the tourniquet, to allow of a little blood to reach the bandages. The blood coming in contact with the iron undergoes a chemical change, and forms a thick, adhesive mass, which closes the lips of the wound, and excludes all contact of air. Shortly afterward I remove the tourniquet, when no hemorrhage can take place, owing to complete closure of the wound and through compression over the veins and arteries. To ensure the latter effect more thoroughly, I previously envelop the limb up to the elbow with rollers of bandage firmly and moderately placed from below upward. As regards the use of a tourniquet, perhaps it would be better still to substitute Esmarch's elastic bandages.

The points of practical importance gained by the method I submit are the following. The wound heals by first intention; the healthy living tissues uniting without suppuration, or, in other words, no "putrefactive fermentation" takes place, just the same condition—the aseptic—as claimed for Professor Lister's method; the non-use of ligatures and sutures a frequent cause of septic mischief; and last, though not least, its simplicity and astonishing results.

I confess I have had few opportunities of testing the merits of my plan of treatment, but the whole process is based upon such scientific principles that I dare hope that it will be essayed by many, and the result honestly reported.

—*Med. and Surg. Rep.*

EXOPTHALMIA TREATED BY GALVINIZATION

In the *Gaceta Medica Italiana*, Dr. D'Ancona relates the case of a woman, aged nineteen, suffering for two years, the symptoms being well marked.

In spite of all kinds of treatment she had arrived at such a stage of cachexia that her life was despaired of. At length galvanization with ten elements of Stohrer's portable battery was tried; and on finding that it was followed by rapid signs of amelioration, it was persevered in for five months. During this time one hundred seances, lasting from three to five minutes each, were given to the patient. She gained thirty pounds in weight; her face lost its paleness, and regained its natural color; the exopthalmia disappeared almost completely, as well as the enlargement of the thyroid body, and the pulse fell from 130 to 90, menstruation was restored, and in every respect the health of the patient was entirely re-established.—*Medical & Surgical Reporter.*

SYPHILIS TRANSMITTED BY VACCINATION.

It is reported that in a small village near Frankfurt en the Oder, twenty-six children were vaccinated from a vaccinifer, subsequently found to be the victim of hereditary syphilis. Twelve are said to have escaped infection, while the remainder suffered from constitutional disease.—*Gazz. Med. Ital. Lomb.*

OVARIOTOMY.

A correspondent of the *Clinic* writes that in the Samaritan Hospital no cases are received except those of ovarian tumor. About twenty beds are provided and every operation is done in the room in which the patient is to lie. The patient is fastened down to the operating table by a strap over the knees and by both hands being strapped firmly so that no movement can occur. The anæsthetic used is bichloride of mythelin. A rather free incision is made, the contents of the cyst drawn off by large trocar, until the tumor can be withdrawn, when the pedicle is tied by two or more catgut ligatures the tumor removed and the pedicle dropped back into the cavity. The incision is closed by interrupted sutures in the usual way, and antiseptic gauze applied. It is commonly necessary to dress the wound but twice. If the case does well the dressing is removed about the fifth day, when the wound is generally found to be united. In two weeks the patient may be about the ward.

TREATMENT OE FAVUS.

Sawicki uses a paste of pulverized chalk or gypsum containing 5-10 per cent. of carbolic acid. This is applied all over the head after cutting the hair short. On the third day the dressing is removed, the head washed with soft soap and water, and the paste reapplied. A little oil may be added to render the dressing more pliable. It is said to effect a cure after three or four applications.

—*Przeglad Lekarski Krakoski.*

MURIATE OF CALCIUM IN TUBERCULOSIS.

This remedy possesses a most wonderful power in controlling, if not actually curing, many forms of tubercular disease. In my experience, I have found no remedy on which so much reliance can be placed in tuberculosis as on this salt; more especially, however, this remark applies to the wasting diseases of children. It has been most extensively used by me during the past four years, and with the most gratifying results—having prescribed it in every form of tubercular disease that has come before me during this period.—ROBERT BELL, F. R. C. P., in London *Lancet.*

Dr. Bell has used it successfully in pulmonary consumption and in glandular and bone scrofula, as well as in tabes mesenterica, and in tubercular peritonitis. Dose for adults, 20 grs., more or less, after meals. It requires to be perseveringly used, and Dr. Bell advises nutrition in conjunction with it; the inunction of olive oil is also recommended. —*Louisville Med. News ; London Lancet.*

OPERATIVE TREATMENT OF INTERNAL PILES.

Mr. Annadale discusses the comparative advantages of the clamp and cautery, and the ligature in the operation for internal piles, in the Edinburg *Medical Journal* for June, 1877. He claims for the former the following advantages:

1. By means of the clamp and cautery the piles are at once removed, and do not remain in the rectum as dead and putrid masses.
2. The irritation and pain are not so severe or so prolonged as in the operation by ligature.
3. The patient's confinement to bed and to the house is much shorter.
4. The resulting sores heal more quickly, and are attended with less risk of suppuration, and its attendant local and general dangers.—*Med. Record.*

CHUCHILL'S TINCTURE OF IODINE.

The following is Churchill's formula as given in the fifth edition of his Diseases of Women. He stated then, 1864, that he had been using it for twenty years:

℞. Iodin. pur....................oz. iiss.
Iodid. potassi....................oz. ss.
Spt. rectificat....................f oz. xii.
Alcohol....................f oz. iv. Solve.

After employing this tincture for thirteen years, I know no single agent in the local treatment of uterine disorders at all equal to it. It may be used as a stimulant, alterative, counter-irritant, caustic, and as a hemostatic, and for the purpose of exciting absorption of hypertrophied tissue. Its hemostatic properties are of especial utility in the treatment of hemorrhagic endometritis, and after the use of the curette or forceps in the removal of smaller intra-uterine growths, hypertrophies of the glandular and vascular elements of the lining membrane.—*American Practitioner.*

TONSILITIS.

My experience in the treatment of this disease with the remedies recommended in the books has been such as to cause me to lay them all on the shelf, except aconite. I have treated quite a number of cases, and I am free to confess that I never saw any decidedly beneficial results from the usual remedies, as from the use of gargles. In this disease I desire to see some improvement in the course of twenty-four hours, but I never have when treated in the "usual way." In the acute disease I give aconite to control the fever and iodide of baryta 1st., 2 grs., every two hours. A friend to whom I recommended it informs me that he has used it in chronic hypertrophy of the tonsils, in five grain doses, three times a day, with most satisfactory results.—*Ec. Med. Jour.*

MR. AUGUSTUS SALA, the accomplished *litterateur*, bears warm testimony, in the Illustrated London News, to the liberality of the medical profession. He says: "All the stingy people in London seem to have come to the front for the purpose of abusing the doctors because they do not always give dates and items in the accounts which they furnish to their patients, but make instead a certain charge for 'medical attendance.' I own myself that I am somewhat prejudiced in the matter. I have had in my day a great deal to do with the doctors, and I have found them, as a rule, the noblest, the most humane, and the most charitable of mankind. It strikes me very forcibly that, so far from being 'fleeced' by the general practitioner, we are often apt

(unconsciously, of course,) to fleece him by cruelly deferring the payment of his bill. Why should we make him wait six months or a year for his due? He has his rent and taxes, and baker and butcher, to pay, as we have, and very frequently his carriage to keep. Is he to eat lint or stethoscopes, or sustain nature by the hypodermic injection of morphia, or the external exhibition of collodion? We should pay our doctors promptly, and then we should know what they are charging us for."—*Med. News.*

ON THE FREQUENT CONNECTION BETWEEN ECZEMA AND DIABETES MELLITUS.

Braxton Hicks states, that of the women who applied to him on account of eczema of the genitals, about eight or nine out of ten have diabetes mellitus in a decided form. This is readily accounted for, by the irritating action on the skin of saccharine urine, but in these cases eczema of other portions of the body is often present, and is not amenable to the ordinary treatment until the glycosuria has been controlled. He mentions the well-known occurrence of sugar in the urine of patients suffering from carbuncle, and directs attention to this association of diabetes with general eczema.—*Lancet.*

PREGNANCY AND SUCKLING.

Professor Dupal says whenever a woman asks you whether, having become pregnant, she ought to continue to suckle her infant, you should reply in the negative, and advise her to procure a nurse, for you may be certain that disturbances will manifest themselves before long, to the great detriment of the child's health.

TREATMENT OF SHINGLES BY TOPICAL APPLICATIONS OF PERCHLORIDE OF IRON.

Dr. Amedee Mercier speaks highly of the good effects of this method. It consists in painting the zona twice daily with a mixture of thirty grammes of perchloride of iron of the codex and ten grammes of alcohol. M. Mercier (These de Paris, March 2, p. 7,) has arrived at the conclusion that the treatment of zona by topical applications of perchloride of iron gives unvarying results, and that the alcoholic solutions should be used in preference to any other.—*London Med. Record.*

CARBOLATE OF SODA IN THE TREATMENT OF NERVOUS AFFECTIONS OE THE RESPIRATORY PASSAGES.

According to Dr. Pernot, all nervous and spasmodic affections of the bronchi—asthma, catarrh, influenza, and simple colds at the outset—are markedly influenced by the vapor of this raw carbolate of soda. On whooping-cough its action is most striking. He has found, from numerous observations, that after from two to ten days of treatment the paroxysms of coughing become much less frequent, less prolonged, and less severe, the vomiting diminishes or ceases, and the respiration becomes easier. He has never seen the symptoms become worse after his treatment was begun. The treatment consists in volatilizing the liquid by means of heat in the sick-room two or three times a day. About dr. x. of the liquid are placed in a porcelain vessel and exposed to the flame of an alcohol lamp. It volatilizes almost completely. The patient is allowed to breathe a purer atmosphere for two or three hours between each sitting, but a saucer containing some of the liquid is constantly kept under the bed. Dr. Pernot reports a few cases of pertussis and one of asthma, which illustrate well the action of the remedy, and certainly seem to justify all that he claims for it. Where a porcelain cup and an alcohol lamp cannot be obtained, a heated fire-brick may be used to volatilize the drug.—*Lyon Medical,* Sept. 23, 1877.

OIL OF STAVESACRE IN SCABIES.

B. Squire recommends the use of the stavesacre, obtained by expression, as a colorless, odorless and unirritating remedy in the treatment of scabies.

### FRACTURE OF THE FEMUR.

Dr. Louis Bauer has quite a lengthy paper in the St. Louis *Clinical Record* for March on the treatment of fractures of the shaft of the femur, from which we make the following abstract:

The first part of the paper takes the ground that shortening in such fractures is not caused by muscular contractions forcing the lowerfragment over the upper. but by the position of the trunk leaning toward the affected side, forcing the upper fragment over the lower. In support of this opinion he quotes the necessity of support to the arm in fractures of the humerus to prevent the formation of a false joint. He reviewed Prof. Hamilton's method of treatment, and contends that extension is unnecessary. Dr. B. formerly used the wire breeches, but now has adopted a double inclined splint, made of sheet iron, and fashioned to fit the limb frem the crest of the ilium to the foot. He says he has used this in a number of cases without any shortening. Dr. B. highly extols Hogden's apparatus, but considers the extension feature needless.

---

### SARRACENIA PURPUREA IN GOUT.

According to Foucaut the stem and root of Sarracenia purpurea is an effective remedy in chronic form of gout. It is employed in form of an infusion, made from 1 to 2 spoonfuls of the powder at a time, mornings and evenings, and once during the day 1 spoonful made into an infusion as a prophylactic. The violence of the attacks becomes greatly diminished, and the alveolar movements more regular.—*Arch. der Med. in Ph. Zeit. f. Russel.*

---

Dr. Griffith recommends the following application to the ulcerations in the severe and very painful sore-throat of scarlatina: chloral, five grains; glycerine, twenty-five grains. After this has been applied with a brush, the pain is much diminished, and the patient can swallow medicine or food without the severe pain which the action caused before.—*N. Y. Med. Jour.*

### TO BLISTER THE SKIN EXTEMORANEOUSLY.

Into a watch-glass, pill-box, or any similar small receptacle, pour ten drops of concentrated water of ammonia aqua ammonia fortior); cover the liquid with a bit of linen or a bit of cotton wool, and at once apply the cup to the skin where the blister is required. Press so that the vapor is confined to the inside of the vessel. A red circle will directly be observed outside, when it will be certain vesication has taken place. Half a minute or so is all the time required to obtain the result. The blister may be dressed in the usual manner of dealing with a blister from cantharides. Acetic acid, concentrated, applied to the skin, will also in a few minutes produce vesication. In each case evaporation should be prevented by some suitable covering. Bibulous paper slightly wetted with a little of the ethereal extract of cantharides, instantly applied to the skin, and covered with a piece of adhesive plaster, will answer for the same purpose.—*Detroit Lancet.*

---

### WARM BATHS IN TRAUMATIC TETANU

In a communication to the Winer medicinisches Doctoren-Collegium which we find published in the *Allg. Wiener Med. Zeitung*, Dr. Zechmeister gives an account of his treatment of ten cases of traumatic tetanus; six of these, several being very dangerous ones, recovered under his treatment. This consisted in keeping the patient for several (3, 5, and in one case 11) hours in a warm bath, and after a short interval (1–2 hours) repeating the procedure, retaining the patient as long as before. He resorted to no other internal or external treatment. In the course of discussion, it was mentioned that this plan was not a new one as Dr. Gottlieb Kraus had reported cases thus treated in 1869.—*Clinic.*

---

The best local anæsthetic for dental operations is the extract of eucalyptus. Apply one drop on cotton to the sensitive dentine just before excavating.

